# Shoplifting Behavior Among Patients With an Eating Disorder at a Medical Correctional Center in Japan: A Cross-Sectional Study

**DOI:** 10.3389/fpsyt.2022.767170

**Published:** 2022-05-18

**Authors:** Etsuko Miyamoto, Yusuke Okumura, Kazushi Maruo, Seiichi Kitani

**Affiliations:** ^1^Medical Correction Center in East Japan, Akishima-shi, Japan; ^2^Department of Biostatistics, Faculty of Medicine, University of Tsukuba, Tsukuba, Japan

**Keywords:** theft, anorexia, recidivism, eating disorder, shoplifting

## Abstract

**Purpose:**

In Japan, the incarceration of patients with eating disorders (EDs) owing to repeated shoplifting has become a social issue. This study examined the shoplifting behavior of inmates with EDs at our medical correctional center, with the objective of delineating their characteristics, identifying an adequate treatment plan, and preventing recidivism.

**Methods:**

The participants consisted of 32 incarcerated patients with EDs (22 females, 10 males) charged with shoplifting, from a medical correctional center in East Japan. A cross-sectional study was employed. Data were collected by retrieving the patients' medical records and through individual interviews conducted by psychiatrists.

**Results:**

The food-specific shoplifting ED group (those who had never shoplifted anything other than food) had a core pathology of the binge-purge type of anorexia nervosa with juvenile onset (*p* = 0.044). Furthermore, they demonstrated an average gap of 8 years between the onset of ED and their first shoplifting episode. The non-specific shoplifting ED group (those who shoplifted food and other items) typically shoplifted before the onset of ED (*p* = 0.001). They experienced the onset of ED after incarceration (*p* = 0.004) and had comorbid disorders (*p* = 0.024). The food-specific group required a psychosocial approach focusing on maintaining healthy body weight and mental stability, whereas the non-specific group required multiple forms of support for substance abuse and behavioral addiction, as well as interventions to reduce impulsive behavior.

**Conclusion:**

Early intervention is necessary to improve the prognosis of patients with EDs and shoplifting behavior.

## Introduction

The relationship between shoplifting and eating disorders (EDs) has been studied since the 1980s (1–7). Comprehensively, 13–67% of patients with an ED—particularly those exhibiting binge-purge (BP) behavior—have reported engaging in shoplifting, mostly for food (5, 8–10). It remains unclear why those with EDs exhibit shoplifting behavior. However, one reason indicated in a previous study by Brozek et al. was the effect of semi-starvation (11).

In the studies by Goldner et al. (4) and Selby et al. (6), shoplifting behavior was associated with low self-esteem, elevated depressive symptoms, and purging behaviors at the time of the assessment. Shoplifting behavior was related to ED symptomatology (e.g., binge eating, vomiting, laxative use, diuretic use, diet pill use, crash dieting, fasting, and compulsive exercise) and higher total scores on the Eating Disorder Inventory and Health Information Questionnaire.

Asami et al. (8) provided data from 2002 to 2011 regarding 41 patients with EDs and associated shoplifting and 14 with EDs and related drug offenses. The shoplifting group had higher educational achievement and steadier employment. However, their ED symptoms and interpersonal dysfunction were more severe. In the shoplifting group, a significant association between low body weight and obsessive-compulsive behaviors was reported, although no antisocial or impulsive characteristics were recorded, compared with those who were drug offenders.

In addition, a large-scale Swedish cohort study on EDs by Yao et al. (12) revealed that the number of people convicted of theft was 2.5 times and 4.3 times higher for anorexia nervosa (AN) and bulimia nervosa (BN), respectively, compared with the control group.

While previous studies (2, 3, 9) have indicated that shoplifting behavior is affected by low body weight and BP behavior, few (2, 9) have investigated the pathological relationship between shoplifting patterns and ED subtypes, especially between the restricting type of AN and binge-purge type of anorexia nervosa (AN-BP) or between BN and AN-BP.

In 2017, Japan counted 200 female inmates with EDs. This represented 5.1% of the entire female inmate population, more than 80% of whom had been sentenced for years owing to repeated low-value (mostly below 200 USD) food theft. However, the number of male inmates with ED was unverified.

The number of male inmates with EDs was so small that each general prison for men could not identify the actual number of inmates with EDs; thus, the number of male inmates with EDs was unverified and lacked national statistics. Our medical correctional center has experience with only a few male a year inmates with EDs.

Through research conducted at the medical correctional center, we identified two shoplifting behavior patterns in patients with EDs: food-specific behavior (i.e., those who have never shoplifted anything other than food) and non-specific behavior (i.e., those who shoplifted food as well as other items). Most incarcerated patients with EDs, particularly those exhibiting binge eating or laxative abuse, steal food for personal use or to compulsively save it. This suggests a slightly different pattern from kleptomania—an impulse control disorder characterized by repetitive theft—because most incarcerated patients shoplift for personal use, a behavior in line with their ED symptoms. According to Blanco et al. (13), although *shoplifting* and *kleptomania* are sometimes used synonymously, there are significant differences between the two terms. Shoplifting is defined as the act of stealing an item from a store. By contrast, kleptomania is a psychiatric condition characterized by recurrent failure to resist impulses to steal objects unneeded for personal use or their monetary value (13).

This study did not focus on kleptomania, as it is a separate psychiatric condition; instead, only shoplifting was investigated. However, if a patient suffers from an ED for an extended period, the associated compulsive behavior notably shifts from shoplifting to kleptomania.

The Japanese government seeks to reduce the rate of recidivism by implementing the “Plan for Re-Criminal Prevention” program. According to the Japanese annual report on crime (14), ~49,000 adult prisoners, of whom 8.4% are female, are incarcerated in the country's 62 correctional facilities for adults. Although the total number of inmates is decreasing annually, theft is the most common reason for incarceration for both male (32.1%) and female (45.4%) inmates. As evident from these percentages, the number of female inmates arrested for shoplifting is much higher than that of males.

During the study period, between September 2012 and December 2016, only a few older inmates (those aged 60–70 years) with EDs were staying at our correctional center. However, since 2019, the number of older inmates has increased. Some of them were incarcerated for shoplifting and demonstrated partial symptomatology corresponding to an ED. Shoplifting accounts for 85% of theft crimes for inmates aged 65 and over and 52.4% for those under the age of 65. Additionally, 30% of inmates aged 65 and over, and 25% of inmates under the age of 65, who have been arrested for a theft crime, reoffend within 2 years. They may be incarcerated for up to 3 years for repeated shoplifting, even if the value of the shoplifted items is low, which results in prisons being occupied by older inmates who shoplift repeatedly.

This study aimed to define the typical characteristics of patients with EDs incarcerated for shoplifting and examine the pathological behavioral differences in various shoplifting patterns to create an individual preventive practice against recidivism. Understanding these patterns and characteristics may help develop targeted interventions to improve incarcerated patients' health and reduce recidivism.

## Materials and Methods

### Participants

This study analyzed all inmates with an ED at a medical correctional center in East Japan, the largest national correctional hospital operated by Japan's Ministry of Justice. Inmates with an ED who exhibited significant weight loss and could not adjust to life in general prisons were transferred to our medical correctional center. Between September 2012 and December 2016, 38 patients with EDs with low body weight who needed immediate treatment were transferred. We examined these 38 patients (28 women, 10 men; 28% and 13% of all psychiatric patients, respectively). Licensed psychiatrists had diagnosed these patients with an ED as defined by the diagnostic criteria in the Diagnostic and Statistical Manual of Mental Disorders, fifth edition (15). The Research Ethics Committee of the Medical Correction Center in East Japan (approval number 30-3) approved the study design. However, the requirement for informed consent was waived by the committee owing to the study's retrospective nature.

### Design

A cross-sectional study design was employed, where we obtained participants' clinical information, including age of onset of the disorder, age at transfer to correctional center, body mass index (BMI), comorbidity, length of stay, and duration of illness, from their medical records and through individual weekly interviews conducted by psychiatrists (primarily, the first author).

Data regarding the participants' family history, educational background, marital status, shoplifting pattern, frequency of incarceration, engagement in multiple offenses, occurrences of shoplifting episodes, and other sociodemographic and incarceration-related variables were collected from the judicial records that had already been gathered from all inmates at the time of incarceration.

The first author was employed as a full-time psychiatrist at the medical correctional center during the study period and continues to work there as the chief psychiatrist (8 years in total, until 2021). The first author interviewed all participants once a week throughout their stay period (average duration of stay: 8–10 months, see Table 1). In addition, we conducted a separate program for ~30% of the participants every week for a total of 8 weeks. The program required participants to reflect on their life since childhood, to describe life events on a graph, including weight gain and loss, and to learn about the impact of these events. The program also required them to recall their ED symptoms and the subsequent impact on shoplifting behavior and to think about ways to prevent recidivism. During the interview and program, special attention was paid to the onset of purging, shoplifted items, shoplifting episodes, date of the first shoplifting episode, and adverse experiences in childhood and adolescence missing from their criminal and medical records. This was done because the participants typically hid their shoplifting behavior and adverse experiences for several years, even decades. Individuals with ED often had no memory of their own medical history or were in denial about it and had poor knowledge of their ED, which typically had an onset of 8 to 20 years earlier. However, after establishing a trustworthy relationship with the psychiatrist during the consultation, the patients gradually began to describe their experiences from their childhood and adolescence.

**Table 1:** Comparison of characteristics between food-specific shoplifting and non-specific shoplifting groups.

**Characteristic (Continuous variables)**	**Food-specific (*n* = 20) *Mean (SD), range***	**Non-specific (*n* = 12) *Mean (SD), range***	***p*-value**	***d***
Age of transfer^#^ (year)	42.5 (12.8), 24–73	41.9 (9.4), 23–54	0.874	0.05
Body mass index (kg/m^2^)	12.7 (1.5), 9.9–15.1	13.9 (2.2), 10.2–17.5	0.120	0.66
Age of ED onset (years)	20.9 (6.6), 14–40	29.6 (11.4), 15–48	0.028*	1.01
ED duration (years)	21.6 (10.9), 5–43	12.3 (13.4), 0.5–39	0.054	0.79
Interval between ED onset and first shoplifting conviction (years)	8.6 (7.6), 0–30	−2.4 (15.5), −24–28 [1]	0.047*	0.98
Age of first shoplifting conviction (years)	29.5 (10.8), 15–54	27.0 (12.4), 15–46 [1]	0.582	0.22
Frequency of incarceration (times)	1.6 (0.8), 1–4	2.5 (2.0), 1–6	0.157	0.66
Characteristic (Binary variables)	Proportion (%)	Proportion (%)	*p*-value	*h*
Female	75.0	58.3	0.483	0.45
Binge-purge type of anorexia nervosa	100.0	75.0	0.044*	1.45
Onset after incarceration	0.0	41.6	0.004**	0.86
Comorbidity	50.0 [2]	91.6	0.024*	1.27
Multiple offenses	0.0	41.6	0.004**	0.86
Shoplifting before onset	0.0	54.5 [1]	0.001**	1.15
Unmarried status	57.8 [1]	30.0 [2]	0.245	0.63
≥ High school degree	80.0	63.6 [1]	0.405	0.48

Welch's t-test for the continuous items, and Fisher's exact test for the binary items; *p < 0.05, **p < 0.01; ED, eating disorder, SD, standard deviation, d, absolute value of Cohen's d, h, absolute value of Cohen's h, [.], number of missing values; ^#^ entry in correctional facilities in Japan for specific medical reasons.

### Data Analysis and Comparison of Shoplifting Patterns

Comparative analyses were conducted to investigate the relationship between shoplifting behavior and EDs. We first divided the participants into two groups based on their shoplifting behavior: food-specific shoplifting (those who have never shoplifted anything other than food items in their life) and non-specific shoplifting (those who have shoplifted any non-food items [cosmetics, stationery, daily necessities, books, jewelry, and others] at least once in their life, regardless of whether they have ever stolen food or not). We then compared these groups using Welch's *t*-test and Cohen's *d* for continuous items (age of ED onset, duration of the disorder, interval between ED onset and first shoplifting episode, age at first shoplifting episode, and frequency of incarceration) considering their unequal variance and sample sizes. Subsequently, Fisher's exact test and Cohen's *h* were used to examine cross-group differences for the binary items (the percentages of inmates who were female and unmarried, had a high school education or above, had AN-BP, showed onset of an ED after incarceration, had a comorbid disorder, exhibited multiple offenses, and had shoplifted before the onset of the ED). Owing to the small proportion of missing data, we performed complete-case analyses for univariable analyses.

Furthermore, we also applied a multivariable model to the binary outcome of non-specific shoplifting. First, the missing data were imputed using the multivariate imputation by chained equations (MICE) method (16), with an imputation rate of 100. Next, we implemented a multiple logistic regression model, including the abovementioned continuous and binary variables, as the covariates for Firth's adjustment (17, 18). We performed the backward variable selection with the criteria of *p* = 0.20 and also selected variables with more than 50% imputed data sets (19).

SPSS Statistics 25.0 for Windows (IBM Corp., Armonk, NY, USA) and R software (R Core Team, Vienna, Austria) were used for all statistical analyses.

## Results

### From the Individual Interviews

During our weekly interview, we found that most inmates had been underweight (BMI 14–17 kg/m^2^) for many years and had experienced repeated episodes of purging or laxative abuse before entering a correctional center. In addition, more than 20% of the participants and their families were unaware of the disease and had never visited a medical institution or received any treatment before incarceration. Incarcerated patients with EDs tended to have a higher level of education (a high school education or above), following a pattern similar to that typically observed in a general population of patients with EDs (20, 21). Most participants reported being raised by pressuring families who prioritized education and learning. As teenagers, participants described having strived to have met their parents' expectations, and most of them had little knowledge of EDs and denied being underweight.

Participants had strong interpersonal relationship problems and poor social skills, which might have increased their chances of self-isolation, avoidance of medical consultations, and refusal of help from neighbors or friends. After being incarcerated and before being transferred to our medical facility, inmates' ED behaviors typically worsened, and they lost more weight owing to stress caused by the strict rules and living conditions in prison.

Over the course of their lives, some shoplifters moved from food-specific to non-specific shoplifting. Some inmates began shoplifting by stealing food only and then gradually widened the variety of stolen items to include non-food ones. The more frequently they shoplifted, the greater their loss of control, as shoplifting became more of a compulsion. This gradual change along the spectrum could explain their transition from food-specific to non-specific shoplifting.

### Comparison of Shoplifting Patterns

There were a total of 114 inmates in the psychiatric ward. We examined all the 32 (20 food specific and 12 non-specific) participants who had committed ED-related shoplifting. Table 1 provides a comparison of the sociodemographic and criminal characteristics between the food-specific and non-specific shoplifting groups. All the participants in the food-specific shoplifting group had AN-BP and tended to have experienced juvenile onset with core pathology. None of these individuals showed onset after incarceration, and none of them shoplifted before the onset of their ED. Furthermore, among the food-specific shoplifting group, an average of 8.6 years lapsed between the onset of ED and first occurrence of shoplifting, with an average of 21.6 years passing between the onset of ED and imprisonment.

By contrast, the non-specific shoplifting group had a significantly greater tendency to exhibit onset after incarceration, to present comorbidities, to have committed multiple offenses other than theft, and to have committed shoplifting before the onset of ED (see Table 1 for further details). This group tended to shoplift regardless of changes in their medical condition, and this tendency was more prominent in male participants. We observed no significant differences between the groups in terms of the EDs' mean duration, age of first shoplifting conviction, frequency of incarceration, percentage of female inmates, unmarried status, and a high school education or above.

As shown in Table 2, the logistic regression analysis included the variables of comorbidity, multiple offenses, interval between the onset of ED and first shoplifting conviction, and marital status. Subsequently, a significant difference was found for interval between the onset of ED and first shoplifting conviction (*p* = 0.042).

**Table 2:** Multiple logistic regression for the outcome variable of food-specific or non-specific shoplifting groups.

**Variable**	**Odds ratio**	**Lower CL**	**Upper CL**	***p*-value**
Comorbidity	16.87	0.83	343.18	0.066
Multiple offenses	20.90	0.78	556.62	0.070
Interval between ED onset and first shoplifting conviction (years)	33.67	1.14	992.49	0.042*
Unmarried status	0.12	0.01	1.93	0.134

Lower and upper CL: 95% lower and upper confidence limits. *p < 0.05 Large odds ratios were estimated due to the complete separations.

## Discussion

A strong association can be observed between shoplifting behavior and EDs (5, 7, 22). Most previous research have investigated EDs in young individuals. However, the inmates we examined exhibited chronic and severe symptomatology as they were older and had suffered from the disorder for a longer period. This suggests that some EDs are not being diagnosed and treated at an early stage. When shoplifting patterns were compared, we found significant differences between the food-specific and non-specific shoplifting groups, with the results showing that the food-specific shoplifting group had a core pathology of AN-BP.

Changes in medical condition and shoplifting frequency were also correlated. The frequency of food shoplifting increased when the inmates lost a substantial amount of weight, which may reflect starvation (11). Goldner et al. (4) and Selby et al. (6) ascertained that current shoplifting was related to ED symptomatology (binge eating, vomiting, laxative use, diuretic use, diet pill use, crash dieting, fasting, and compulsive exercise), higher Early Development Instrument total scores, and higher High Intelligence Quotient total scores. These findings provide support for our hypothesis which states that shoplifting behavior because of mental dysfunction is associated with the starvation that often accompanies severe EDs. When the patients' symptoms improved, and stress decreased, the shoplifting behavior also decreased. The more frequently they were incarcerated, the more often ED behaviors (e.g., purging, hyperactivity, food waste) manifested recurrently. Thus, a psychosocial approach focusing on maintaining the inmates' weight and psycho-physical stability might be effective in reducing their shoplifting behavior. Asami et al. (8) stated that supportive treatment should be considered for inmates with EDs. Our results also support this notion, especially for the food-specific shoplifting group with AN-BP core pathology.

On the contrary, the non-specific shoplifting group was more likely to engage in multiple offenses, shoplift before the onset of their disease, show ED onset after incarceration, and present comorbid conditions. Table 1 indicates that comorbidity and multiple offenses are the two significant variables in the univariate analysis, and they are also selected in the logistic regression, as seen in Table 2. Moreover, interval between the onset of ED and first shoplifting conviction were also found to be significant in the logistic regression. The evidentiary power of these variables is high on the non-specific group. The members of this group tended to shoplift regardless of any change in their medical condition. Thus, for these patients, specific treatments should be provided focusing on their impulsivity, substance abuse, or behavioral addiction, which may all lead to recidivism.

When we considered the reasons behind high incarcerations among ED shoplifters in Japan, we examined the effects of legal, social, and cultural factors. First, there is no criminal law specific to shoplifting. Therefore, when shoplifting is committed during a probation period, even after receiving a fine, recidivist shoplifters are incarcerated for years regardless of the value of the stolen items. Second, there is only a small number of facilities specializing in the treatment of EDs. It is difficult for general practitioners, families, and individuals to identify the presence of an ED at an early stage. Third, due to cultural reasons, when individuals initially shoplift, they are unlikely to confess to their behavior and, therefore, do not discuss the same with medical staff or their families. Finally, over a prolonged period, such behavior tends to become chronic and severe.

According to Yao et al. (12), it is common to request a criminal record screening for individuals with a mental illness. However, it is not typically requested for those with an ED, owing to a false perception that only adolescents have EDs and that they do not commit crimes, or to a general disregard for EDs as a serious mental illness that warrants medical care. Yao et al. (12) conclude that medical professionals should request criminal record screenings for all mental illnesses, including EDs, particularly because EDs and their association with shoplifting behaviors have the best prognosis with early intervention. Our results also support this notion, regardless of the patient's gender. In other words, early diagnosis and treatment of EDs are especially important to prevent recidivism.

At the onset stage of EDs, medical staff, public health providers, school officials, and other professionals should consider not only the possibility of comorbid mental disorders, such as personality disorders, alcohol or drug dependence, and developmental disorders (23–25), but also the possibilities of shoplifting behaviors or addictions emerging. Based on the characteristics of shoplifting patterns among patients with EDs, consistent responses and support are required throughout the criminal justice process, from the initial visit with a referee and subsequent imprisonment, parole, and probation, to the community hospital visit.

### Limitations

Owing to the small sample size, it is difficult to generalize the findings of this study. Furthermore, since the participants are inmates who have been incarcerated for an extended period, it is difficult to generalize these results to present-day patients with EDs who shoplift. In addition, the subtypes of EDs may not reflect the characteristics of all incarcerated patients with EDs in Japanese prisons. Another limitation is the lack of standardized assessments of interpersonal relationship problems, social skills, self-isolation, and avoidance of medical consultations. Moreover, since this research is exploratory in nature, many statistical tests were performed without accounting for multiplicity. Therefore, the results of statistical tests need to be cautiously interpreted. This study also offers limited information regarding the effectiveness of preventive measures against recidivism, leaving room for further research. Comparative studies across samples in different prisons are also needed to provide a more comprehensive view of the problem.

## Conclusion

We investigated the characteristics of two groups of inmates specified by their shoplifting behavior: food-specific and non-specific shoplifting. Treatment for patients who engage in food-specific shoplifting may require the maintenance of a healthy body weight and mental stability, while treatment for those who engage in non-specific shoplifting may require many forms of support and a multifaceted intervention by a multidisciplinary team for substance and behavioral addictions and impulsivity. Early intervention is necessary to improve the prognosis of patients with EDs and shoplifting behavior.

## Data Availability Statement

Publicly available datasets were analyzed in this study. This data can be found here: The data that support the findings of this study are publicly available in the White Paper on Crime (http://hakusyo1.moj.go.jp/en/nendo_nfm.html). Restrictions apply to the availability of these data, which were used under license for this study. Data are available at http://www.moj.go.jp/ENGLISH/index.html with the permission of the Ministry of Justice in Japan.

## Ethics Statement

The study design was approved by the Research Ethics Committee of the Medical Correction Center in East Japan (approval number 30-3). However, the requirement for informed consent was waived by the committee owing to the retrospective design of the study. Written informed consent for participation was not required for this study in accordance with the national legislation and the institutional requirements.

## Author Contributions

SK, YO, and EM performed material preparation, data collection, and analysis. The first draft of the manuscript was written by EM. All authors contributed to the study conception and design, commented on previous versions of the manuscript, read, and approved the final manuscript.

## Conflict of Interest

The authors declare that the research was conducted in the absence of any commercial or financial relationships that could be construed as a potential conflict of interest.

## Publisher's Note

All claims expressed in this article are solely those of the authors and do not necessarily represent those of their affiliated organizations, or those of the publisher, the editors and the reviewers. Any product that may be evaluated in this article, or claim that may be made by its manufacturer, is not guaranteed or endorsed by the publisher.
